# The Puf-Family RNA-Binding Protein Puf2 Controls Sporozoite Conversion to Liver Stages in the Malaria Parasite

**DOI:** 10.1371/journal.pone.0019860

**Published:** 2011-05-18

**Authors:** Katja Müller, Kai Matuschewski, Olivier Silvie

**Affiliations:** Max Planck Institute for Infection Biology, Parasitology Unit, Berlin, Germany; Agency for Science, Technology and Research (A*STAR), Singapore

## Abstract

Malaria is a vector-borne infectious disease caused by unicellular, obligate intracellular parasites of the genus *Plasmodium*. During host switch the malaria parasite employs specialized latent stages that colonize the new host environment. Previous work has established that gametocytes, sexually differentiated stages that are taken up by the mosquito vector, control expression of genes required for mosquito colonization by translational repression. Sexual parasite development is controlled by a DEAD-box RNA helicase of the DDX6 family, termed DOZI. Latency of sporozoites, the transmission stage injected during an infectious blood meal, is controlled by the eIF2alpha kinase IK2, a general inhibitor of protein synthesis. Whether RNA-binding proteins participate in translational regulation in sporozoites remains to be studied. Here, we investigated the roles of two RNA-binding proteins of the Puf-family, *Plasmodium* Puf1 and Puf2, during sporozoite stage conversion. Our data reveal that, in the rodent malaria parasite *P. berghei*, *Puf2* participates in the regulation of *IK2* and inhibits premature sporozoite transformation. Inside mosquito salivary glands *puf2(-)* sporozoites transform over time to round forms resembling early intra-hepatic stages. As a result, mutant parasites display strong defects in initiating a malaria infection. In contrast, *Puf1* is dispensable *in vivo* throughout the entire *Plasmodium* life cycle. Our findings support the notion of a central role for *Puf2* in parasite latency during switch between the insect and mammalian hosts.

## Introduction

*Plasmodium* parasites, the causative agents of malaria, are transmitted by female *Anopheles* mosquitoes. During the probing phase prior to the blood meal, sporozoites are injected into the skin of the mammalian host [1]. The motile sporozoites actively migrate in the skin, enter the peripheral blood circulation, and then rapidly reach the liver. Sporozoites invade hepatocytes by forming a parasitophorous vacuole (PV) [2], where they transform into replicative exo-erythrocytic forms (EEFs). After intense multiplication during 2–6 days, depending on the *Plasmodium* species, mature EEFs release thousands of merozoites, which invade erythrocytes and initiate the pathogenic blood stage cycle [3].

*Plasmodium* sporozoites are formed inside oocysts in the mosquito midgut, but become fully infective only after colonization of the insect salivary glands. This maturation process is associated with the up-regulation of a specific subset of genes, referred to as Up-regulated in Infective Sporozoites (*UIS*) genes [4]. Regulation of gene expression in *Plasmodium* remains poorly understood. Genome sequencing data initially revealed a paucity of specific transcription factors in *Plasmodium* [5]. Recently however, a family of genes related to the plant Apetala-2 (AP2) transcription factors has been identified in *Plasmodium* and related apicomplexan parasites [6], [7], and proposed to play a central role during life cycle progression. Molecular genetic studies have demonstrated vital roles of two stage-specific AP2 factors in *Plasmodium berghei*, a rodent malaria parasite widely used as a model [8], [9]. One of these factors, the AP2-Sp transcription factor, is required during sporozoite differentiation and binds to a specific DNA sequence found in the promoter region of many genes expressed in sporozoites, including, but not restricted to, *UIS* genes [8]. Intriguingly, genes containing AP2-Sp binding sites are associated with a wide range of biological processes, such as sporozoite formation, host cell invasion or liver stage development. This observation strongly suggests that additional mechanisms participate in the fine-tuning of gene expression during sporozoite development and stage conversion. Another factor, called SLARP or SAP1, controls the expression of a subset of genes in sporozoites, and plays a critical role during intrahepatic development of the parasite [10], [11]. It is still unclear whether SLARP/SAP1 acts on a transcriptional or a post-transcriptional level. The cellular localization of SLARP/SAP1 remains controversial [10], [11], and the absence of any domain known to bind nucleic acids suggests an indirect role.

More recently, Zhang and colleagues reported that the protein kinase IK2, initially termed UIS1 [4], controls global gene expression in sporozoites at a post-transcriptional level [12]. IK2 phosphorylates the translation initiation factor eIF2alpha and down-regulates protein synthesis [12], [13]. *P. berghei* lacking *UIS1/IK2* display a partial loss of infectivity associated with premature transformation of sporozoites in the mosquito salivary glands [12]. The contribution of RNA-binding prxoteins in translational regulation has not been studied in sporozoites yet, but has been well characterized in *Plasmodium* sexual stages. In female gametocytes, many transcripts encoding ookinete proteins are translationally repressed by a DEAD-box RNA helicase called DOZI, which binds to the 3′ untranslated region (UTR) of target mRNAs such as *P28* and blocks their translation until occurrence of gamete fertilization and differentiation into a zygote and ookinete [14], [15]. Whether DOZI plays a role in sporozoites is not known, but other RNA-binding proteins may participate in translational regulation in sporozoites, including members of the Puf-family.

Puf proteins are evolutionary conserved in eukaryotes and are characterized by the presence of a RNA-binding Puf domain, named after the *Drosophila melanogaster* protein Pumilio and the *Caenorhabditis elegans* protein fem-3 binding factor (FBF), and consisting of eight imperfect repeats of 36 amino acids (PFAM: PF00806) [16], [17]. Puf proteins typically bind to the 3′ UTR of target mRNAs and repress their translation or induce their degradation (reviewed in [18] and [19]). *Plasmodium* parasites possess two genes encoding proteins with Puf domains, *Puf1* and *Puf2* [20]. In *P. falciparum*, both *Puf1* (PFE0935c) and *Puf2* (PFD0825c) are differentially expressed in gametocytes [20], [21]. Targeted gene disruption in *P. falciparum* recently revealed a role of *Pf*Puf2 in repressing gametocytogenesis and male gametocyte differentiation in the human malaria parasite [22]. Whether the Puf2 protein plays additional, perhaps vital, roles in subsequent life cycle stages remains to be shown. Interestingly, microarray data indicate that *Puf2* is most highly expressed in *P. falciparum* sporozoites [23], and in *P. berghei*, expression of both *Puf1* (PBANKA_123350) and *Puf2* (PBANKA_071920) has been reported in sporozoites, where *Puf1* was initially identified as *UIS9* [4], [24]. In this study, we used a reverse genetic approach to investigate the roles of Puf1 and Puf2 in *P. berghei*, with the aim to identify potential mRNA binding proteins that play critical roles in sporozoite stage conversion.

## Results

### Targeted gene deletion of *P. berghei Puf1* and *Puf2*

**Figure 1:**
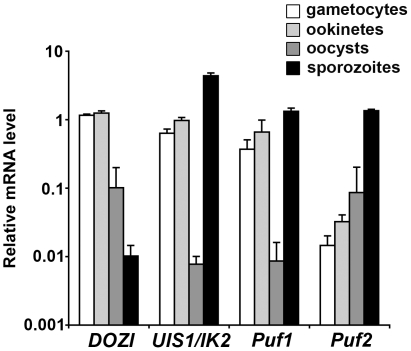
*Puf1* and *Puf2* are upregulated in *P. berghei* sporozoites. Shown is an expression profiling of selected transcripts of RNA regulatory proteins, the DDX6-family DEAD-box helicase *DOZI* [15], the Puf proteins *Puf1* and *Puf2* [20], and of the eIF2alpha kinase *UIS1/IK2* that controls sporozoite latency [12]. *P. berghei* purified gametocytes, ookinetes, oocysts and salivary gland sporozoites were analyzed by RT-qPCR using primers specific for *DOZI*, *UIS1/IK2*, *Puf1* and *Puf2*. Expression data from two independent experiments are shown and were normalized to the level of *GFP* transcripts, which are expressed under the control of the *EF1alpha* promoter [26].

We first assessed the expression of *Puf1* and *Puf2* during *P. berghei* development in the insect vector, in comparison to *DOZI* and *UIS1/IK2*, using quantitative RT-PCR (**Figure 1** **)**. Similarly to *UIS1/IK2* [12], we found that *Puf1* and *Puf2* are upregulated in *P. berghei* salivary gland sporozoites (**Figure 1** **)**. This was expected for *Puf1*, which was initially described as *UIS9* [4], [24]. Furthermore, *Puf1* was also upregulated in gametocytes and ookinetes, similarly to *IK2* and *DOZI*. In good agreement with published microarray data [24], only low levels of *DOZI* mRNA were detected in *P. berghei* sporozoites (**Figure 1**). In contrast to *Puf1* and *Puf2*, *DOZI* steady state mRNA levels were down-regulated in infectious salivary gland-associated sporozoites resulting in ∼100 fold lower levels in the latent transmission stage. Together, the expression profiling indicated that both *Puf* members could play a role in sporozoite stage conversion, as has been described previously for the eIF2alpha kinase *UIS1/IK2* [12].

**Figure 2:**
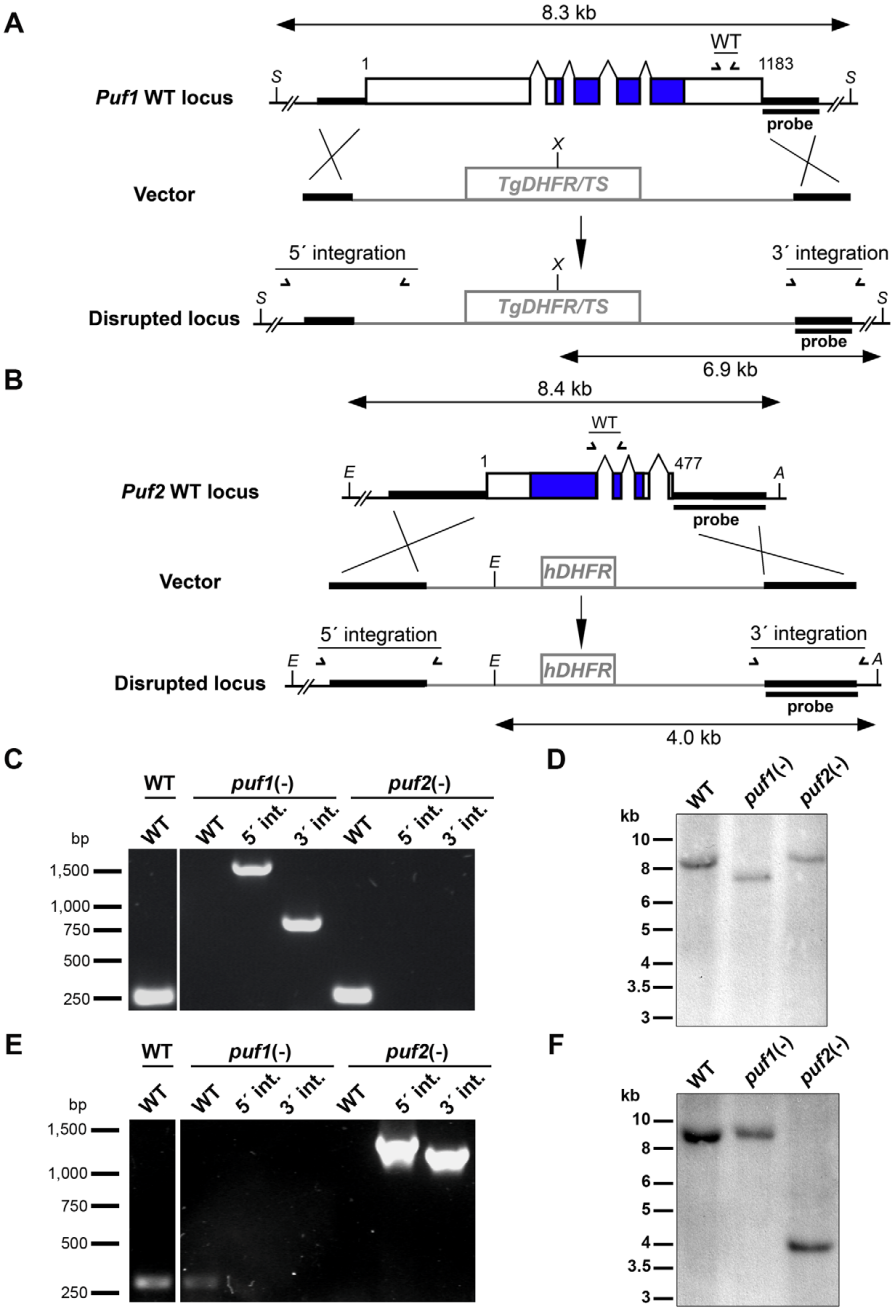
Targeted gene deletion of *Puf1/UIS9* and *Puf2* in *P. berghei*. (A–B) Replacement strategy to generate the *puf1(-)* and *puf2(-)* parasites. *P. berghei PUF1* gene (A) consists of five exons encoding an 1183 amino-acid protein (PBANKA_123350), whereas *PUF2* (B) consists of four exons encoding a 477 amino-acid protein (PBANKA_071920). The PUF domains are shown in blue. For each gene, the wild-type (WT) genomic locus was targeted with a replacement plasmid containing 5′ and 3′ regions of *PUF1* or *PUF2* and a positive selectable marker, *Toxoplasma gondii dhfr/ts* or human *DHFR*, respectively. Upon a double crossover event, the *PUF1* or *PUF2* gene is replaced by the selectable marker. Replacement- and wild type- specific test primer combinations and expected PCR fragments (WT, 5′ integration and 3′ integration) are indicated by arrows and lines, respectively. Restriction sites, Southern probes and expected restriction fragments are also shown. *S, Spe*I; *X, Xho*I; *A, Afe*I; *E*, *Eco*RV. (C) *Puf1* replacement-specific PCR analysis. Confirmation of the predicted gene targeting is achieved by specific primer combinations (5′ and 3′ integration), which can only amplify a signal from the recombinant locus. A wild type-specific PCR reaction confirms the absence of residual wild-type parasites in the clonal *puf1(-)* population. (D) Southern blot analysis of genomic DNA isolated from WT, *puf1(-)* and *puf2(-)* parasites, using digoxigenin-labelled probes specific for *Puf1*. After digest with *Spe*I and *Xho*I, the *Puf1* probe hybridizes to a 8.3 or a 6.9 kb fragment in WT and *puf1(-)* parasites, respectively. (E) *Puf2* replacement-specific PCR analysis. Confirmation of the predicted gene targeting is achieved by specific primer combinations (5′ and 3′ integration), which can only amplify a signal from the recombinant locus. A wild type-specific PCR reaction confirms the absence of residual wild-type parasites in the clonal *puf2(-)* population. (F) Southern blot analysis of genomic DNA isolated from WT, *puf1(-)* and *puf2(-)* parasites, using digoxigenin-labelled probes specific for *Puf2*. After digest with *Afe*I and *Eco*RV, the *Puf2* probe hybridizes to a 8.4 kb fragment in WT and a 4.0 kb fragment in *puf2(-)* parasites.

In order to investigate the functional importance of *Puf1/UIS9* and *Puf2* in *P. berghei*, we generated loss-of-function mutants (**Figure 2**). We used a replacement strategy to disrupt the endogenous *Puf1* (**Figure 2A**) or *Puf2* (**Figure 2B**) gene copy by double crossover homologous recombination [25]. Targeting constructs containing 5′ and 3′ fragments of either *Puf1* or *Puf2* flanking a pyrimethamine-resistance cassette were used to transfect *P. berghei* parasites that constitutively express GFP (ANKA cl507) [26]. Recombinant parasites were selected with pyrimethamine in the mouse drinking water, and cloned by limiting dilutions. For both genes we were successful in generating clonal knockout parasite populations, as demonstrated by PCR and Southern blot analysis of genomic DNA (**Figures 2C**–**F**). For *Puf2* we also generated a second independent knockout clone, which was phenotypically identical to the first *puf2(-)* clonal parasite line (unpublished data). This indicates that *Puf1* and *Puf2* do not play any vital role during *P. berghei* erythrocytic stages, in good agreement with successful generation of *Pfpuf2(-)* parasites [22].

### *puf1*(-) and *puf2*(-) parasites produce gametocytes that develop to sporozoites in mosquitoes

**Figure 3:**
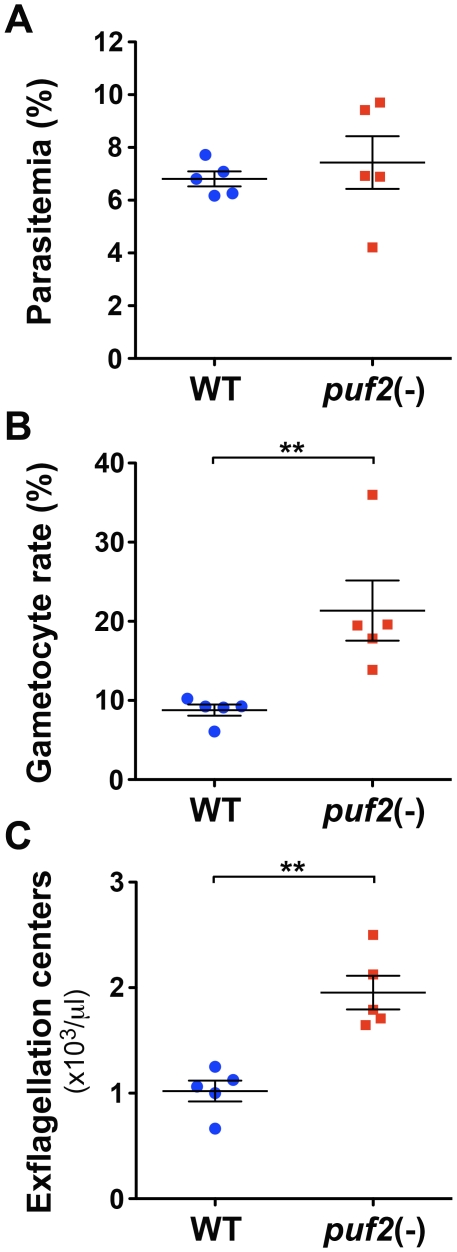
Gametocytogenesis is increased in puf2(-) parasites. Groups of C57BL/6 mice (n = 5) were injected intravenously with 107 WT or puf2(-) infected erythrocytes. Blood was collected from the mice 4 days later to determine the parasitemia (A), the proportion of gametocytes among parasites (B), and the number of exflagellation centers per µl of blood (C). Results are expressed as mean +/− SEM. **, *p*<0.01 (Mann-Whitney test).

*puf1*(-) and *puf2*(-) parasites were indistinguishable from WT parasites in development and growth of asexual blood stages and produced gametocytes. Because *Pf*Puf2 has been shown to control gametocytogenesis in *P. falciparum* [22], we analyzed in more detail the sexual development of *P. berghei puf2*(-) parasites. After injection of 10^7^ infected erythrocytes intravenously into groups of five C57BL/6 mice, parasitemia at day 4 were similar in mice infected with WT or *puf2*(-) (**Figure 3A**). However, the proportion of gametocytes among all parasite stages was significantly higher in *puf2(-)* than in WT parasites (**Figure 3B**). We then examined the ability of mature male gametocytes to exflagellate in *puf2*(-) parasites. The number of exflagellation centers in mouse blood was significantly higher for *puf2*(-) parasites than for WT parasites (**Figure 3C**), suggesting that male gametocytes contribute to the increased gametocytogenesis in *Pbpuf2*(-) parasites, in full support of the data reported for *P. falciparum Puf2-*deficient parasites [22].

After transmission to *Anopheles stephensi* mosquitoes, both *puf1*(-) and *puf2*(-) parasite lines produced oocysts and high numbers of sporozoites (**Table 1**). The number of *puf2*(-) oocysts was significantly higher than for WT, consistent with the higher gametocyte rates. Intriguingly, we found lower numbers of oocysts and salivary gland sporozoites in *puf1*(-)-infected mosquitoes, as compared to WT parasites (**Table 1**). Although the differences were not statistically significant, we cannot exclude an effect of *puf1* depletion on oocyst development and sporogony.

**Table 1:** Loss of infectivity of *puf2(-)* sporozoites in C57BL/6 mice.

Parasites	Number of oocysts/mosquito^a^ (mean ± SD)	Number of salivary gland sporozoites/mosquito^a^ (mean ± SD)	Route of injection^b^	Number of infected/Number of injected	Prepatency period (days)^c^
WT	182 (± 164)	31,600 (± 18,600)	bites (d 21)	3/3	3
			i.v. (d 18)	2/2	3
			i.v. (d 21)	6/6	3.5
			i.v. (d 25)	4/4	3
*puf1(-)*	137 (± 100)	11,400 (± 4,700)	bites (d 17)	3/3	3
			i.v. (d 21)	6/6	3.5
*puf2(-)*	320 (± 234)^d^	25,000 (± 18,800)	bites (d 21)	3/4	(5)
			i.v. (d 18)	2/4	(5)
			i.v. (d 25)	0/4	NA

a The number of midgut oocysts and salivary gland sporozoites was determined at d10–14 and d18–25, respectively, after the infectious blood meal, from at least three independent feeding experiments.

b C57BL/6 mice were exposed to the bites of 10 infected mosquitoes or injected intravenously (i.v.) with 1,000 sporozoites, 18–25 days after mosquito infection.

c The prepatent period is defined as the number of days after sporozoite inoculation until detection of infected erythrocytes by microscopic blood smear examination. Brackets indicate that not all animals became infected. NA, not applicable.

d p<0.05 in comparison to WT, as determined by Kruskal-Wallis followed by Dunn's test.

### Liver infection is impaired in *Puf2*-deficient parasites

We then analysed the infectivity of *puf1*(-) and *puf2*(-) sporozoites to susceptible mice. C57BL/6 mice were injected intravenously with 1,000 WT, *puf1*(-) or *puf2*(-) *P. berghei* sporozoites, or exposed to the bites of 10 infected mosquitoes, the natural transmission route (**Table 1**). Emergence of erythrocytic stages, resulting from complete liver stage development, was monitored by microscopic examination of daily blood smears. With both inoculation routes, all mice injected with *puf1*(-) sporozoites developed a parasitemia, with no delay as compared to WT parasites (**Table 1**). In contrast, only a fraction of the mice injected with *puf2*(-) sporozoites developed a parasitemia, with a two-day delay as compared to WT, indicative of at least 100-fold reduction of infectivity (**Table 1**). Moreover, *puf2*(-) sporozoites isolated late after mosquito infection (at day 25) were not capable of inducing a blood stage infection in mice.

**Figure 4:**
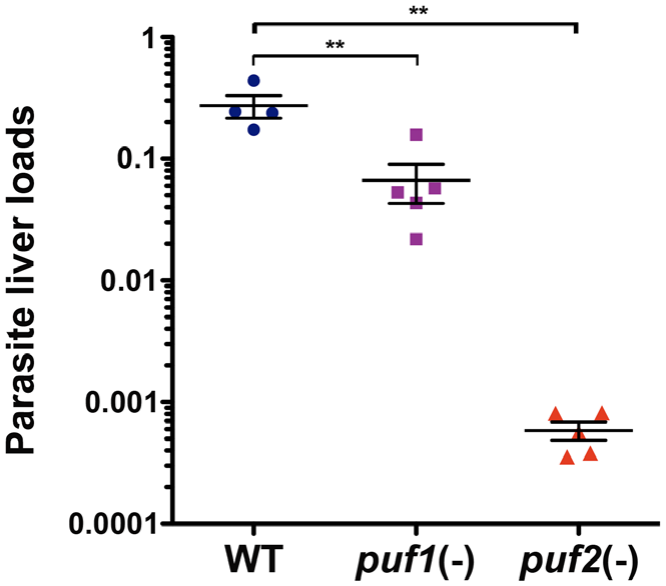
Liver infection is severely impaired in *puf2(-)* parasites. Parasite loads were determined by RT-qPCR analysis of mouse livers (n = 4 or 5 per group) harvested 42 hours after intravenous injection of 10,000 WT, *puf1(-)* or *puf2(-)* sporozoites. Results are expressed as the relative expression of *Pb*18S normalized to mouse GAPDH. The means +/− SEM are indicated by lines **, *p*<0.01 (Mann-Whitney test).

We next injected C57BL/6 mice intravenously with WT, *puf1*(-) or *puf2*(-) sporozoites isolated on day 18 from mosquito salivary glands. Forty-two hours after infection, livers were removed and the parasite loads were quantified by RT-qPCR. As shown in **Figure 4**, the *puf2(-)* liver loads were extremely reduced (∼500 fold) as compared to WT, confirming that infectivity of *puf2(-)* sporozoites to C57BL/6 mice is severely impaired. The reduction of parasite liver loads as measured by RT-qPCR is consistent with the delay or absence of parasitemia in mice injected with *puf2*(-) sporozoites (**Table 1**), therefore we assume that the absence of *Puf2* did not interfere with 18S rRNA quantification. Interestingly, we also observed a significant, although less pronounced (∼4 fold), reduction of *puf1(-)* parasite liver loads (**Figure 4**). Our findings demonstrate that *PbPuf2* plays an important *in vivo* role only in the pre-erythrocytic phase of the *Plasmodium* life cycle. In contrast, *Puf1/UIS9* appears to be dispensable for parasite life cycle progression, at least under the conditions tested.

**Figure 5:**
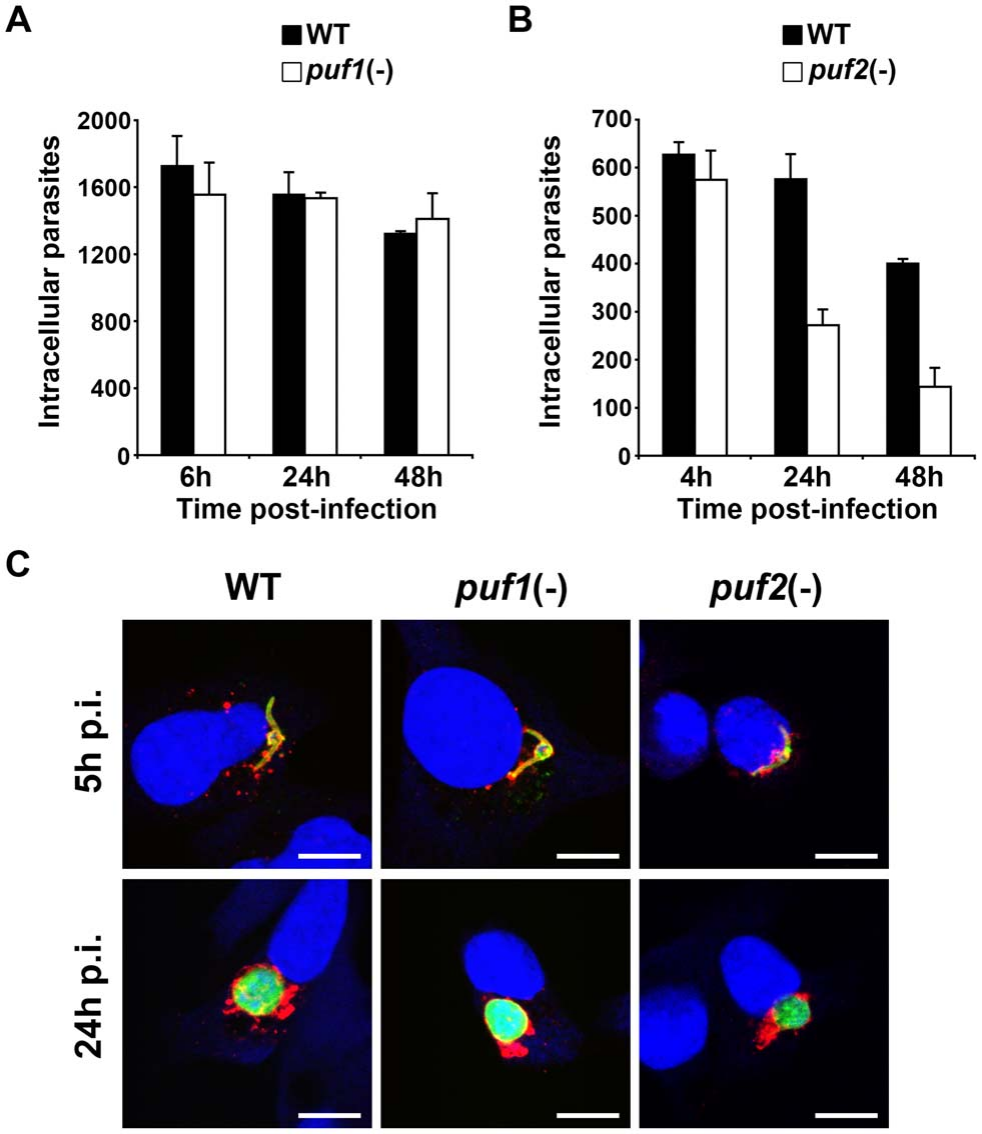
*puf2(-)* sporozoites are infective *in vitro*. (A) HepG2 cells were infected with WT or *puf1(-)* sporozoites and the numbers of infected cells were determined at 6, 24 and 48 h post-infection (p.i.). Results are expressed as the mean number of infected cells in triplicate wells +/− SD. (B) HepG2 cells were infected with WT or *puf2(-)* sporozoites and the numbers of infected cells were determined at 4, 24 and 48 h post-infection (p.i.). Results are expressed as the mean number of infected cells in triplicate wells +/− SD. (C) Confocal microscopy analysis of HepG2 cells cultured for 5 and 24 hours post-infection (p.i.) with WT, *puf1(-)* or *puf2(-)* sporozoites, using antibodies against UIS4 (red), CSP (5 h p.i., green) or HSP70 (24 h p.i., green). Nuclei were stained with DRAQ5 (blue). Bars, 10 µm.

We also determined the *In vitro* infectivity of *puf1(-)* and *puf2(-)* sporozoites isolated on day 22 from mosquito salivary glands, in cultured HepG2 hepatoma cells (**Figure 5**). Both *puf1(-)* and *puf2(-)* sporozoites entered hepatoma cells as efficiently as WT, as evidenced by similar numbers of infected cells at early time points (4–6 hours) (**Figure 5A**). While the number of EEFs at later time points (24–48 hours) was similar in WT- and *puf1(-)*-infected cultures (**Figure 5A**), it was reduced in the case of *puf2(-)* parasites (**Figure 5B**). Whereas early after infection a vast majority (81% ±3%; n = 122) of intracellular WT sporozoites expressed UIS4, a transmembrane protein that localizes to the membrane of the PV [27], only half of *puf2(-)* parasites were stained with UIS4 antibodies (53% ±9%; n = 127). This indicates that a substantial fraction of *puf2(-)* sporozoites failed to form and/or remodel the PV *in vitro*, which probably explains the reduced EEF numbers quantified at later time points. In addition, we cannot exclude a moderate impairment during liver stage development in *puf2(-)* parasites, as suggested by the reduction of EEF numbers observed between 24 and 48 hours post-infection *in vitro*. Nevertheless, most *puf2(-)* sporozoites that formed a PV and expressed UIS4 were capable of developing into EEFs like WT and *puf1(-)* parasites (**Figure 5C**). Taken together, our data indicate that *Puf2* plays a critical role during transmission of *P. berghei* sporozoites to the mammalian host, but is not required for liver stage development *per se*.

### *puf2(-)* sporozoites transform prematurely in the mosquito

**Figure 6:**
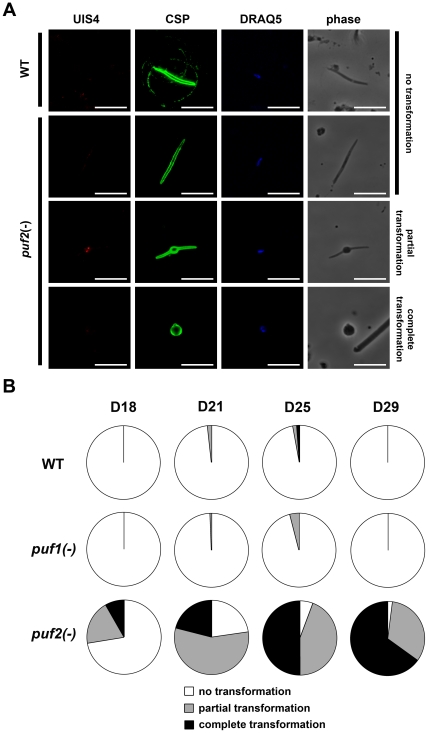
Premature transformation of *puf2(-)* sporozoites in the mosquito. (A) Fluorescence microscopy of WT and *puf2(-)* sporozoites isolated from mosquito salivary glands 25 days after infection, and labelled with anti-UIS4 (red) and anti-CSP (green) antibodies. Nuclei were stained with DRAQ5 (blue). Bars, 10 µm. (B) The proportion of non-transformed, partially transformed and fully transformed sporozoites was determined by microscopic examination of sporozoites isolated from mosquito salivary glands 18, 21, 25 or 29 days after infection with WT, *puf1(-)* or *puf2(-)* parasites.

*In vivo* data suggested that, over time, *Puf2*-knockout sporozoites rapidly loose infectivity in the mosquito (**Table 1**). To better characterize this phenomenon, we carefully analyzed *puf2(-)* sporozoite development in the mosquito (**Figure 6**). Strikingly, we observed that a major proportion of *puf2(-)* sporozoites showed signs of premature transformation, characterized by a bulb-like aspect or even complete rounding-up (**Figure 6A**). In WT parasites, transformation of sporozoites is typically observed at 37°C in culture medium, irrespective of the presence of host cells [28]. In *puf2(-)*-infected mosquitoes, however, the proportion of transformed sporozoites increased over time during the course of infection in the mosquitoes, which are kept at 20°C (**Figure 6B**). Quantification of partial and complete transformation in all three parasite populations revealed that at day 29 almost all *puf2(-)* sporozoites had transformed, whereas only a minor fraction of WT and *puf1(-)* sporozoites exhibited signs of premature transformation (**Figure 6B**). Interestingly, we did not observe expression of the liver stage marker UIS4 or nuclear divisions, as seen in EEFs (Figure 5C), in the transformed *puf2(-)* sporozoites (**Figure 6A**).

### *puf2(-)* sporozoites have reduced levels of *Puf1* and *UIS1/IK2* mRNA

**Figure 7:**
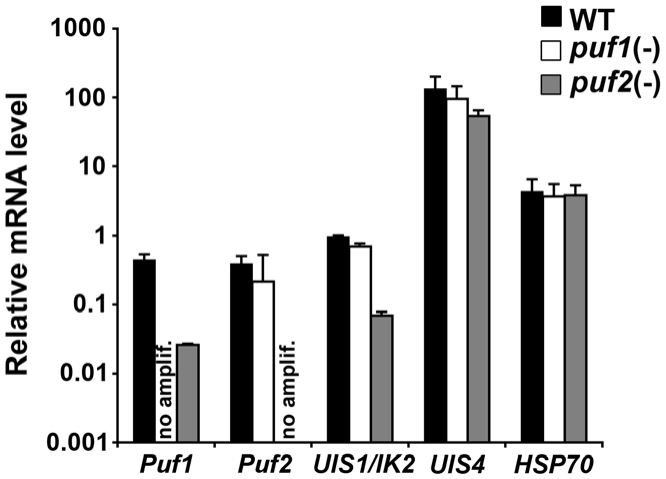
*puf2(-)* sporozoites have reduced levels of *Puf1* and *UIS1/IK2* mRNA. Shown is an expression profiling by RT-qPCR analysis of *Puf1*, *Puf2*, *UIS1/IK2*, *UIS4* and *HSP70* mRNA levels in WT, *puf1(-)* and *puf2(-) P. berghei* sporozoites. Expression data from three independent experiments are shown and were normalized to the level of *GFP* transcripts, which are expressed under the control of the EF1alpha promoter [26]. no amplif., no amplification.

The phenotype of *puf2(-)* parasites is essentially identical to that of parasites that contain a targeted deletion of the kinase *UIS1/IK2* [12]. Similarly to *puf2(-)* parasites, *ik2*(-) sporozoites transform prematurely in the mosquito salivary glands and have a decreased infectivity *in vivo* but not *in vitro* [12]. Therefore, we sought to test expression of *IK2* in *puf2(-)* sporozoites, in comparison to WT and *puf1(-)* sporozoites, using RT-qPCR. As expected from gene deletion, no *Puf1* and *Puf2* mRNA were detected in *puf1(-)* and *puf2(-)* sporozoites, respectively (**Figure 7**). Whereas expression of *UIS1/IK2* was not modified in *puf1(-)* sporozoites, we observed a ∼14 fold reduction of *UIS1/IK2* mRNA in *Puf2*-deficient sporozoites as compared to WT (**Figure 7**). Additionally, we found a ∼17 fold reduction of *Puf1* transcript levels in *puf2(-)* parasites. Conversely, *Puf2* transcript levels were not affected in the absence of *Puf1* (**Figure 7**). As controls, *UIS4* and *HSP70* mRNA levels were similar in the mutant and WT sporozoites. Altogether, these data indicate that *Puf2* regulates a subset of genes in *P. berghei* sporozoites, including *Puf1* and the kinase *UIS1/IK2*. The latter probably explains, at least in part, why the phenotype of *puf2(-)* sporozoites recapitulates that of *IK2*-knockout parasites.

## Discussion

*Plasmodium* sporozoites must persist and remain infectious within the salivary glands of the mosquito for many days until they are eventually transmitted to a mammalian host. Inside the warm-blooded host they need to quickly leave the site of deposition in order to travel to the liver, invade hepatocytes and differentiate into liver stages [29]. The transient developmental arrest of sporozoites inside mosquito salivary glands, termed latency [12], implies efficient control mechanisms to prevent premature transformation before transmission and during transmigration before reaching a suitable host cell. In this study, we identified a factor controlling sporozoite latency in *P. berghei,* the RNA-binding protein Puf2. In the absence of *Puf2*, sporozoites transform prematurely in the mosquito, resulting in a severe loss of infectivity.

Sporozoite conversion into liver stages requires initial remodelling of the parasite pellicle, with disassembly of the inner membrane complex (IMC) and appearance of a bulb that progressively enlarges until the initially elongated sporozoite has transformed into a round form [28], [30]. Previous work has shown that transformation of salivary gland sporozoites is induced at 37°C in culture medium, irrespective of the presence of host cells [28]. It should be noted that differentiation into EEFs involves additional events, including expression of liver stage specific proteins, onset of nuclear divisions and parasite growth. None of these events are observed in axenic culture conditions [28], where instead extracellular sporozoites die rapidly after transformation [31].

We show that *puf2(-)* sporozoites transform prematurely in the mosquito salivary glands, as evidenced by the characteristic bulb-like structures and rounding-up of the parasites. Premature transformation probably impairs the sporozoite functions that depend on IMC integrity, such as parasite motility, cell traversal and invasion, thus resulting in a loss of infectivity. In the absence of *Puf2*, the proportion of transformed salivary gland sporozoites increases over time, which correlates with a progressive loss of infectivity to mice. Interestingly, although most *puf2(-)* sporozoites eventually transform into completely round forms, these forms do not progress to EEF differentiation, as shown by minimal expression of the liver stage marker UIS4 and absence of nuclear division or growth. In contrast, normal differentiation of *puf2(-)* parasites is observed once sporozoites invade cultured hepatoma cells. Collectively, these data strongly suggest that Puf2 plays a major role in preventing premature remodelling of the sporozoites prior to liver infection, but is not required for EEF differentiation.

The defects observed in *puf2(-)* parasites are reminiscent of those described in *IK2*-knockout parasites [12]. Both *puf2(-)* and *ik2*(-) sporozoites transform prematurely in the mosquito and display greatly reduced infectivity to mice. However, both loss-of-function mutants are able to invade and differentiate into EEFs in cultured cells *in vitro,* indicating that they do not play any essential role after host cell infection. These observations, combined with a major down-regulation of *IK2* expression in *puf2(-)* sporozoites, suggest that the phenotype of *Puf2*-deficient parasites can be explained, to a large extent, by *IK2* depletion.

How IK2 prevents sporozoite transformation has yet to be determined. Phosphorylation of the alpha subunit of eIF2 by distinct kinases, such as *Plasmodium* IK2, is a central mechanism in stress-induced translational regulation [32], including in protozoans. For example, the eIF2alpha kinase IK1 regulates responses to starvation stress in *P. falciparum* blood stages [13], and in *Toxoplasma gondii*, phosphorylation of eIF2alpha promotes survival of extracellular tachyzoites [33]. Our data corroborate the findings of Zhang *et al*. [12], which together suggest that a similar stress response operates in sporozoites to maintain them in a quiescent stage.

The founding member of the Puf family, *Drosophila melanogaster* Pumilio (*Dm*PUM), regulates, amongst other functions, abdominal development in the fly *via* translational repression of the maternally inherited hunchback (*hb*) mRNA [34]. The Puf domain of *Dm*PUM binds to a nanos response element (NRE) sequence located in the 3′ UTR of *hb* mRNA. Biochemical data, such as *in vitro* binding assays using recombinant Puf domains expressed in bacteria and heterologous *in vivo* studies using the yeast three-hybrid system, have demonstrated intrinsic binding activity of the *P. falciparum Pf*Puf1 and *Pf*Puf2 to the NRE sequence [20], [21]. Signature RNA sequences that are recognized by the Puf domain vary between species and members of the Puf family, but typically contain a UGUR motif [35], [36], [37], [38]. A large number of *Plasmodium* genes contain UGUR motifs in their 3′ UTR, but their functional significance remains uncertain, especially in the context of the exceptional AT-richness of the *Plasmodium* genome. Therefore, endogenous targets of *Plasmodium* Puf proteins still remain elusive. The *P. falciparum* [22] and *P. berghei* (this study) mutants now constitute potential tools to identify Puf2 target genes in *Plasmodium*. In sporozoites, Puf2 regulates at least two other genes in addition to *IK2* and *Puf1*. Indeed, using RT-qPCR, we found a 4-fold reduction of *Spect* and *Spect2* mRNA levels in *puf2*(-) sporozoites, whereas actin and *AMA1* were not affected (unpublished data). Reduced expression of *Spect* and *Spect2* genes, which are both essential for sporozoite cell traversal and migration to the liver [29], [39], [40], may also contribute to the loss of infectivity of *puf2*(-) parasites *in vivo*.

Whereas Puf proteins typically modulate target mRNA expression by either promoting mRNA turnover or translational repression, they can also activate gene expression or control mRNA subcellular localization (reviewed in [18] and [19]). Our results are not compatible with a role of Puf2 in repressing *IK2*, because *puf2(-)* and *ik2(-)* share a similar phenotype. Puf2 may instead participate in stabilization of *IK2* transcripts. Alternatively, depletion of *IK2* mRNA in *puf2(-)* could be an indirect effect due to activation of an upstream factor that regulates *IK2*.

Whereas *Drosophila* encodes only *Dm*PUM, many organisms, including *C. elegans*, contain two or more genes encoding Puf proteins, which can fulfil partly redundant functions [41]. Therefore, presence of two *Puf* genes in the *Plasmodium* genome might be explained by overlapping or distinct roles. However, our molecular genetics data clearly exclude a vital role for *Puf1* under normal conditions throughout the *P. berghei* life cycle. *Puf1* may be critical under specific conditions, similarly to the role of IK1 in *P. falciparum* during starvation-induced stress [13]. Puf2 might compensate for the absence of Puf1 in *puf1(-)* parasites, but not *vice versa*. While Puf1 in principle might be able to functionally complement for *Puf2* function, depletion of *Puf1* at the mRNA level precludes a hypothetical functional overlap *in vivo.* In this regard, it should be noted that *P. berghei* Puf1 and Puf2 proteins are very different in size (1183 versus 477 amino acids, respectively), and share only little homology (∼27% identity) restricted to the Puf domains.

*P. falciparum* parasites that lack *Puf2* show increased gametocyte rates and a bias towards male gametocytes [22]. These observations fit with the proposed unifying, and perhaps ancestral, role of Pufs in promoting cell proliferation and repressing differentiation [18]. Our findings that Puf2 inhibits sporozoite transformation further support the notion of a central role in suppression of cellular differentiation. Because of the published data from *P.falciparum puf2(-)* parasites we did not investigate sexual development and differentiation of *Pbpuf2(-)* parasites in great detail other than to confirm the previous findings, i.e. an increase in gametocytogenesis in *puf2(-)* parasites, partly due to increased male gametocyte differentiation. In the previous study, life cycle progression of *Pfpuf2(-)* parasites beyond gametocytogenesis was not analyzed [22]. Based on our results in the rodent malaria model system, we predict that *P. falciparum* sporozoites lacking *Puf2* will present a similar phenotype, that is premature sporozoite transformation in the mosquito and decreased infectivity. Therefore, our findings might be of considerable interest in the context of development of genetically attenuated parasites for vaccination [42].

In conclusion, we show here that Puf2 plays a major role in controlling sporozoite latency during host switch, possibly through the regulation of IK2. Our results also highlight the functional importance of post-transcriptional regulation of gene expression during transmission of the malaria parasite between hosts.

## Materials and Methods

### Ethics statement

All animal work was conducted in accordance with the German ‘Tierschutzgesetz in der Fassung vom 18. Mai 2006 (BGBl. I S. 1207)', which implements the directive 86/609/EEC from the European Union and the European Convention for the protection of vertebrate animals used for experimental and other scientific purposes. The protocol was approved by the ethics committee of MPI-IB and the Berlin state authorities (LAGeSo Reg# G0469/09).

### Experimental animals, parasites and cell lines

Female NMRI and C57BL/6 mice were from Charles River Laboratories. We used *P. berghei* ANKA clone 507 parasites, which constitutively express the green fluorescent protein (GFP) [26]. HepG2 cells (ATCC HB-8065) were cultured as described [43].

### *P. berghei Puf1* and *Puf2* gene deletion

A targeting construct for *Puf1* gene knockout was generated by inserting a 503-bp 5′ fragment and a 575-bp 3′ fragment on either side of a *T. gondii DHFR/TS* expression cassette. A construct for *Puf2* gene knockout was generated by inserting a 1001-bp 5′ fragment and a 945-bp 3′ fragment on either side of a human *DHFR* expression cassette. Oligonucleotide sequences are indicated in Table S1. *P. berghei* parasites were transfected with linearized plasmids, using the Nucleofector® device (Amaxa GmbH) as described [44], injected intravenously into naïve NMRI mice, and selected by pyrimethamine treatment in the drinking water. Clonal parasite populations were obtained by limiting dilution series and intravenous injection of one parasite in 10 recipient NMRI mice. One *puf1(-)* and two *puf2(-)* clonal parasite lines were established and phenotypically characterized. Genotyping of WT and recombinant parasites was performed by PCR and Southern blot analysis of genomic DNA. Standard Southern blot analysis was performed using the PCR DIG Probe synthesis kit and the DIG Luminescent Detection kit (Roche), according to the manufacturer's instructions.

### Real time quantitative RT-PCR

Parasite total RNA was extracted with the RNeasy kit (Qiagen) and reverse transcribed with the RETROScript kit (Ambion). Real time PCR was performed on cDNA preparations as described [11], using the StepOnePlus™ Real-Time PCR System and Power SYBR® Green PCR Master Mix (Applied Biosystems), according to the manufacturer's instructions. Expression data were normalized using the constitutively expressed *GFP* gene.

### Immunofluorescence

Parasites were fixed in 4% paraformaldehyde (PFA) and permeabilized with 1% Triton X-100. Immunofluorescence was then carried out using previously described monoclonal antibodies against *P. berghei* CSP [45] and HSP70 [46]. Polyclonal anti-UIS4 antibodies were raised in rabbits immunized with two synthetic peptides from *P. berghei* UIS4 (CLFTDEHKDEINDNIV and CNNVYNMENKSFGPYI) (Eurogentec). DRAQ5 (Biostatus) was used to stain nuclei. Confocal pictures were obtained with a Leica TCS-SP microscope equipped with appropriate filters, and processed with Photoshop software (Adobe Inc.).

### Parasite growth and sexual development

C57BL/6 mice (n = 5) were injected intravenously with 10^7^ infected erythrocytes. Four days later, the parasitemia was determined by microscopic examination of Giemsa-stained blood smears. To analyse exflagellation of male gametocytes, five microliters of tail blood were diluted 1∶25 in RPMI 1640 containing 10% FCS and 50 µM xanthurenic acid, and adjusted to pH 8.0. After 12 min incubation at room temperature, exflagellation centers were counted in a Neubauer chamber. Mean parasitemia and gametocyte rates were compared using the Mann-Whitney non-parametric test. After parasite transmission to *Anopheles stephensi* mosquitoes, the numbers of midgut oocysts and salivary gland sporozoites were determined at day 10–14 and day 18–25, respectively, and compared using the Kruskal-Wallis followed by Dunn's multiple comparison tests.

### Analysis of sporozoite *in vivo* infectivity

C57BL/6 mice were injected intravenously with 1,000 WT or mutant sporozoites isolated from the salivary glands of infected mosquitoes, or exposed to 10 infected mosquito bites, as indicated. Infection was then monitored daily by examination of Giemsa-stained blood smears. The delay of patency was defined as the time before detection of at least one erythrocytic stage in the smears. For quantification of parasite liver loads by real time RT-PCR, C57BL/6 mice were infected intravenously with 10,000 sporozoites. At 42 hours post-infection, livers were harvested, total RNA was extracted with the RNeasy kit (Qiagen) and cDNA synthesized with the RETROScript kit (Ambion). Real-time PCR was then performed with the StepOnePlus™ Real-Time PCR System and Power SYBR® Green PCR Master Mix (Applied Biosystems), using primers specific for *P. berghei* 18S rRNA and mouse GAPDH, as described [47]. Liver parasite loads were compared using the Mann-Whitney non-parametric test.

## Supporting Information

Table S1 **List of oligonucleotides used in this study.**
(PDF)
