# Supplementary material for: The Puf-Family RNA-Binding Protein Puf2 Controls Sporozoite Conversion to Liver Stages in the Malaria Parasite
Source: PLoS One. 2011 May 18;6(5):e19860. doi: 10.1371/journal.pone.0019860 (PMC3097211; doi:10.1371/journal.pone.0019860)
Supplement: Table S1 — List of oligonucleotides used in this study. (PDF) [file pone.0019860.s001.pdf]

# The Puf-family RNA-binding protein Puf2 controls sporozoite conversion to liver stages in the malaria parasite

Katja Müller, Kai Matuschewski, and Olivier Silvie

**Supplemental Table S1.** List of oligonucleotides used in this study.

| Experiment | Oligonucleotide name | Sequence 5´ → 3´ |
|---|---|---|
| **RT-qPCR** | *Puf1* forward | TTATGATAAATGGACTACTAAAAAAAGG |
| | *Puf1* reverse | TTTCCAAGCAATTCTTTTACCTGAG |
| | *Puf2* forward | TTATCTTCTCACAAATATGCCTGC |
| | *Puf2* reverse | ATTTCCCTCAGGAATGTCGTCAAC |
| | *GFP* forward | GATGGAAGCGTTCAACTAGCAGACC |
| | *GFP* reverse | AGCTGTTACAAACTCAAGAAGGACC |
| | *DOZI* forward | TGTCGCAAACACATCGAAATCGTG |
| | *DOZI* reverse | ACCCTAAGTGACCATATCTTCCTG |
| | *UIS1/IK2* forward | GAAAAGTATAAGAATAAGTTTGTTAGTC |
| | *UIS1/IK2* reverse | GATTTATCCTGAACAATATGAATTCC |
| | *UIS4* forward | CCAAACCAAGCGATCATACATACAG |
| | *UIS4* reverse | CTTCACCCACTAAATCGCTTAATTC |
| | *HSP70* forward | AAGAAGCTGAAGCTGTATGCTCTCC |
| | *HSP70* reverse | AGTTCATACCTCCTGGCATTCCTCC |
| | *Pb*18S forward | AAGCATTAAATAAAGCGAATACATCCTTAC |
| | *Pb*18S reverse | GGAGATTGGTTTTGACGTTTATGTG |
| | mGAPDH forward | TGAGGCCGGTGCTGAGTATGTCG |
| | mGAPDH reverse | CCACAGTCTTCTGGGTGGCAGTG |
| ***Puf1* gene disruption** | 5´ fragment forward | CCGGAATTCCTTCCCATTCCCTATTCACCAAAAACC |
| | 5´ fragment reverse | AGGCATATGGGGTAATGCTACTCTATAATTCGGTGG |
| | 3´ fragment forward | ACCCAAGCTTGTGAAAGTACTACAAATAATTGTGGG |
| | 3´ fragment reverse | CGGGGTACCCGTTACAAATATATCTTCTATTATCGCC |
| | WT test forward | AACCCGAATTAACAAAACTTGTAGAAGG |
| | WT test reverse | ATTTGGGTAATTTTCTGAACAACTTATCG |
| | 5´ integr. test forward | GCATTACGCACATATATAGAAGTGTTTGAG |
| | 5´ integr. test reverse | GAATGTATTTTGTACTATCCATTTGTATTCC |
| | 3´ integr. test forward | CCGGAATTCCATACACAAACATACAAAAATAAACACC |
| | 3´ integr. test reverse | ACGCTATTATTATCATCTGCATTTTCGC |

| | | |
|---|---|---|
| ***Puf2* gene disruption** | 5´ fragment forward | TCCCCGCGGGGATAAATAAATATAAGGTCTATAACAAG |
| | 5´ fragment reverse | ATAAGAATGCGGCCGCACATTTTCGTTTTGGGTATACTTAAATGTATATATGC |
| | 3´ fragment forward | CCCAAGCTTTATTTTAAGGGCTATTAATAATTTAGAGG |
| | 3´ fragment reverse | CCGCTCGAGAGCTAATTAATTATAGTATGTCATGTAGAG |
| | WT test forward | GAATATTCTGATGACAATGTTAGGCTCC |
| | WT test reverse | AAATTTAATACCCCTCAGGAATGTCGTC |
| | 5´ integr. test forward | TATGCATATAGACAGATTAATATACACC |
| | 5´ integr. test reverse | CAGCTTCAAGTAGTCGGGGATGTCG |
| | 3´ integr. test forward | TGAGCATTTAAAGCACAATATCTAGG |
| | 3´ integr. test reverse | TCTTATCAAAAGAAACAATGGATATGAGTC |
